# Additional value of lateral tissue Doppler imaging in the assessment of diastolic dysfunction among subjects with pseudonormal pattern of mitral inflow

**DOI:** 10.1186/1476-7120-11-31

**Published:** 2013-08-20

**Authors:** Hack-Lyoung Kim, Joo-Hee Zo, Jae-Bin Seo, Woo-Young Chung, Yong-Jin Kim, Sang-Hyun Kim, Myung-A Kim, Dae-Won Sohn

**Affiliations:** 1 Cardiovascular Center, Seoul National University Boramae Medical Center, Seoul, Korea; 2 Cardiovascular Center, Seoul National University Hospital, Seoul, Korea; 3 Department of Internal Medicine, Seoul National University Boramae Medical Center, Seoul National University College of Medicine, 39 Boramae-gil, Dongjak-gu, Seoul 156-707, Korea

**Keywords:** Diastolic dysfunction, Mitral annulus, Pseudonormal pattern, Tissue Doppler imaging, Transthoracic echocardiography

## Abstract

**Background:**

There has been a lack of research on further stratification of subjects who have pseudonormal pattern of mitral inflow. The study aim was to clarify 2 different groups with different diastolic function grades among these subjects using lateral tissue Doppler imaging (TDI).

**Methods:**

A total of 122 consecutive subjects showing pseudonormal pattern of mitral inflow (E/A ≥ 1 and septal e’/a’ < 1) without structural abnormality were prospectively recruited. TDI measurements were performed from both septal and lateral mitral annuli.

**Results:**

Study subjects were stratified according to lateral TDI pattern (e’/a’ < 1 [n = 50] versus e’/a’ ≥ 1 [n = 72]). Subjects with lateral e’/a’ < 1 had higher values of left atrial volume index (LAVI) and E/e’ compared to those for lateral e’/a’ ≥ 1 (*p* < 0.001 for each). Among subjects with lateral e’/a’ ≥ 1, only 9.3% of subjects had grade II diastolic dysfunction, whereas among subjects with lateral e’/a’ < 1, majority of subjects (64.1%) had grade II diastolic dysfunction (*p* < 0.001). Multiple linear regression analysis showed that lateral e’/a’ was independently associated with LAVI (*β* = −0.484, *p* < 0.001), even after adjusting for potential confounders including age, sex, body mass index, hypertension and diabetes.

**Conclusions:**

In subjects without structural abnormality showing E/A ≥ 1 and septal e’/a’ < 1, lateral TDI measurement is useful in the assessment of diastolic dysfunction. Lateral e’/a’ ≥ 1 is a valuable indicator of early diastolic dysfunction but not of advanced diastolic dysfunction in this population.

## Background

Evaluation of left ventricular (LV) diastolic function is an essential component of routine echocardiographic examination. As the clinical significance of heart failure with preserved ejection fraction (HFpEF) has increased [1,2], information on LV diastolic function and filling pressure is of great importance to clinicians [3]. Doppler analysis of mitral inflow and pulmonary vein flow has been the main clinical tool used to evaluate LV diastolic function. However, this analysis has several shortcomings. It is dependent on loading conditions such as volume status and left atrial (LA) pressure, and is difficult to obtain in patients for whom the image qualities are poor. More recently, tissue Doppler imaging (TDI), which is relatively independent of loading conditions and image quality, has been found to be useful in the assessment of LV diastolic function [4,5]. In addition, the ratio between early diastolic mitral inflow (E) and early diastolic mitral annular tissue velocity (e’) was found to correlate well with LV filling pressure, and are now widely used to diagnose diastolic dysfunction [5-7]. Moreover, combined information of mitral inflow and TDI is able to differentiate between normal and pseudonormal diastolic dysfunction [5].

**Figure 1:**
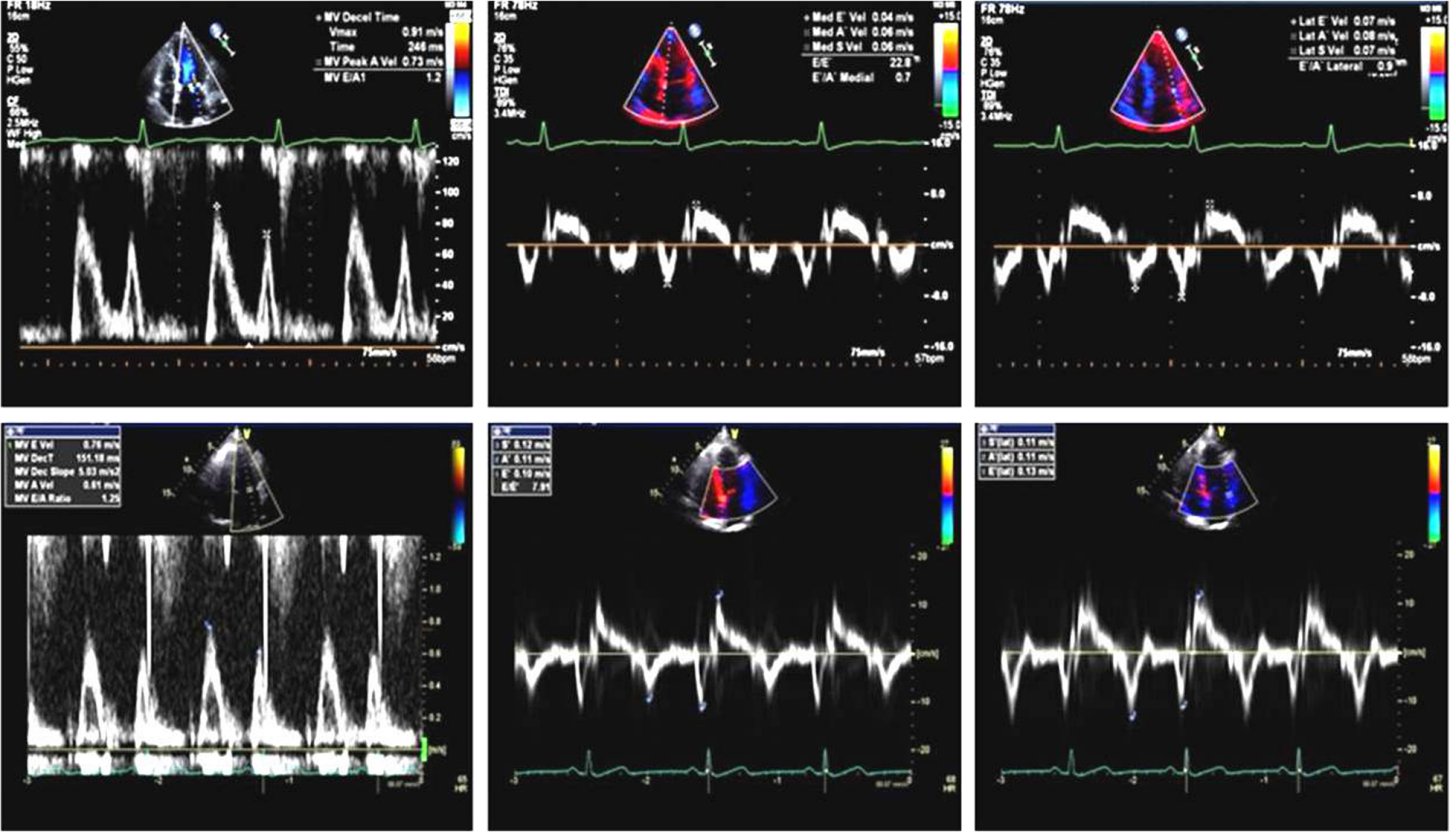
Representative figures of a subject with concordance (upper 3 figures) and discordance pattern (lower 3 figures) of tissue Doppler imaging.

Previously, we have shown that some people have discrepancies in mitral annular velocity patterns between the septal and lateral annulus because the septal annular velocity more rapidly decreases with age, when compared with that of lateral annulus [8]. However, the clinical implications of these findings have not been further studied. The present study focused on subjects with ratios of peak early to late mitral inflow wave velocity [E/A] ≥ 1 and ratios of peak septal early to late diastolic mitral annular tissue velocity [e’/a’] < 1, showing pseudonormal pattern of mitral inflow [5,6]. These subjects are frequently considered to have an advanced stage of diastolic dysfunction. Among these subjects, we have noticed 2 different groups with different severities of diastolic dysfunction according to the lateral TDI patterns: one group with lateral e’/a’ < 1 (concordant to septal TDI) and the other group with lateral e’/a’ ≥ 1 (discordant to septal TDI) (Figure 1). This study describes different characteristics between these 2 groups and suggests additional benefit of measuring lateral TDI in the evaluation of diastolic function in this population.

## Methods

### Study subjects

**Figure 2:**
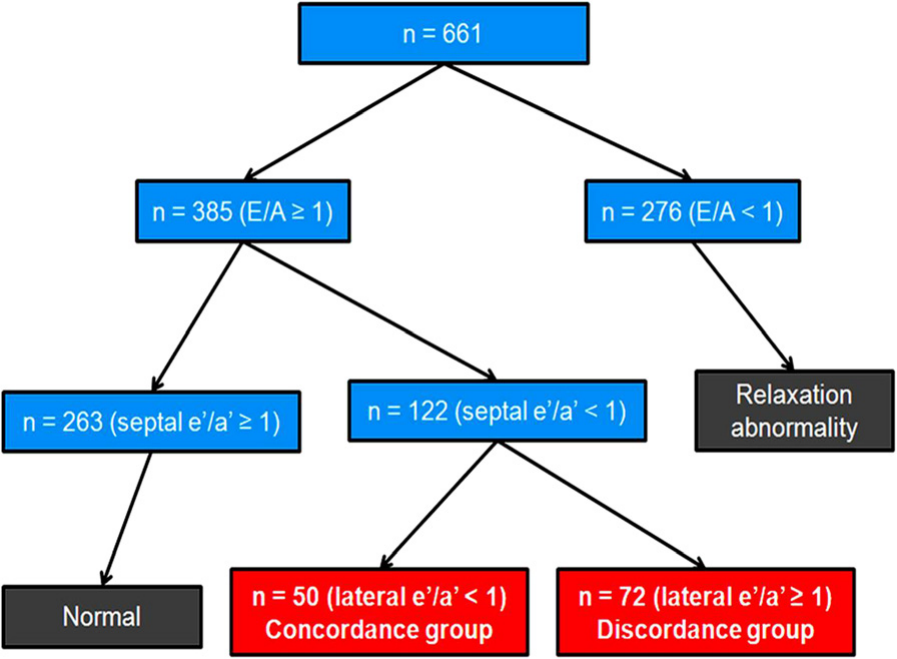
Schematic figures showing enrollment flow of study subjects.

This is a single center study performed at Seoul National University Boramae Medical Center (Seoul, Korea). Between March and October 2012, a total of 661 consecutive subjects in sinus rhythm without significant structural abnormalities in transthoracic echocardiographic (TTE) examination were prospectively recruited. Subjects with LV ejection fraction < 50%, valvular stenosis or regurgitation of more than mild degree, regional wall motion abnormalities, LV hypertrophy, mitral annular calcification, prosthetic heart valve, and intra-cardiac shunt or pericardial effusion were excluded from the study. Subjects with poor sonic window or poor cooperation during the exam were also excluded. These 661 subjects were grouped according to mitral inflow patterns: a group with E/A ≥ 1 (n = 385) and the other group with E/A < 1 (n = 276). Among 385 subjects with E/A ≥ 1, the present study focused on 122 subjects with septal e’/a’ < 1, who were frequently considered to have a pseudonormalization suggesting advanced stage of diastolic dysfunction [5,6]. From clinical experience, we have noticed that these subjects can be stratified into 2 groups with different diastolic dysfunction severities by the lateral TDI pattern. In order to investigate the usefulness of lateral TDI in the assessment of diastolic function, these 122 subjects were then classified according to the pattern of lateral TDI. One group of subjects had lateral e’/a’ < 1, and designated as “concordance (to medial TDI pattern) group” (n = 50). The other group had lateral e’/a’ ≥ 1, and this group was designated as “discordance (to medial TDI pattern) group” (n = 72). Study flow for enrollment is shown in Figure 2. Approval of the study protocol was obtained from the Institutional Review Board of Seoul National University Boramae Medical Center (Seoul, Korea). Informed consent was waived due to the routine nature of the information collected.

### Echocardiography

TTE was performed according to the recommendations of current guidelines [9-11] using commercially available equipments (Sequoia, Siemens Medical Solutions and Vivid 7, GE Medical Systems) in the left lateral decubitus position. M-mode echocardiographic features were used to measure LV and LA dimensions, with LV ejection fraction from the parasternal short axis view at the mitral chordae level. LA volume was calculated using the prolate ellipsoid method [9]. Peak early transmitral filling velocities during early diastole (E), late diastole (A) and deceleration time (DT) were imaged in the apical four chamber view at the tip of mitral leaflets. Color-coded TDI was applied to the apical four chamber view to determine the mean early (e’) and late (a’) velocities at both septal and lateral mitral annuli. Two experienced cardiosonographers performed the echocardiography. Interobserver agreement for lateral E/e’ was evaluated by Spearman’s correlation among 50 subjects. The correlation coefficient was 0.905 in our laboratory.

### LV diastolic function grade

LV diastolic function was graded according to the current guidelines [10]. Normal LV diastolic function was defined as septal e’ ≥ 8 cm/s and LA volume index (LAVI) < 34 ml/m^2^. Grade II diastolic dysfunction was defined as septal e’ < 8 cm/s, LAVI ≥ 34 ml/m^2^, DT 160–200 ms and septal E/e’ ≥ 9. Because we had only enrolled subjects with E/A ≥ 1, there were no subjects with grade I diastolic dysfunction, which is defined as E/A < 0.8. Grade III diastolic dysfunction was defined as septal e’ < 8 cm/s, LAVI ≥ 34 ml/m^2^, E/A ≥ 2, DT < 160 ms and septal E/e’ ≥ 13. There were no subjects with grade III diastolic dysfunction in the study.

### Statistical analysis

Continuous variables were presented as mean ± standard deviation (SD), and categorical variables were expressed as percentages. Continuous variables were compared using the Student *t* test, and categorical variables were compared using the Chi-square test. Pearson’s correlation method was used to assess the relationship between two continuous variables. Multiple linear regression analysis was performed to assess independent association between LAVI and lateral e’/a’ by adjusting for age, sex, body mass index and history of hypertension and diabetes. To identify the best cut-off value of lateral e’/a’ as a predictor of LAVI ≥ 34 ml/m^2^[10], receiver operating characteristic (ROC) curve analysis was used. A *p-*value < 0.05 was considered statistically significant. All statistical analyses were conducted using SPSS 18.0 (Chicago, IL, USA).

## Results

The baseline characteristics of the study subjects are summarized in Table 1. The mean age was 56.0 ± 13.2 years, and 59 (48.4%) subjects were men. The prevalence of hypertension, diabetes and dyslipidemia were 41.8, 13.9 and 15.6%, respectively.

**Table 1:** Comparisons of characteristics among study subjects

**Characteristic**	**All (n = 122)**	**Concordance group (n = 50)**	**Discordance group (n = 72)**	p^*^
Age, years	56.0 ± 13.2	59.6 ± 14.1	53.5 ± 12.0	0.012
Male sex, n (%)	59 (48.4)	23 (46.0)	36 (50.0)	0.664
BMI, kg/m^2^	25.2 ± 4.1	25.7 ± 4.1	24.6 ± 4.2	0.336
Hypertension, n (%)	51 (41.8)	24 (48.0)	27 (37.5)	0.248
Diabetes, n (%)	17 (13.9)	10 (20.0)	7 (9.7)	0.107
Dyslipidemia, n (%)	19 (15.6)	12 (16.7)	7 (14.0)	0.690
Echocardiographic parameters				
LVESD, mm	29.6 ± 3.6	30.7 ± 3.4	28.8 ± 3.5	0.004
LVEDD, mm	48.0 ± 4.3	49.5 ± 3.0	47.4 ± 3.0	< 0.001
LVEF, %	68.1 ± 5.0	67.4 ± 5.8	68.6 ± 4.2	0.213
LVSWT, mm	9.3 ± 1.4	9.5 ± 1.7	9.2 ± 1.3	0.234
LVPWT, mm	9.2 ± 1.3	9.2 ± 1.6	9.1 ± 1.0	0.563
LA dimension, mm	36.7 ± 5.2	39.0 ± 5.3	35.2 ± 4.6	< 0.001
LA volume index, ml/m^2^	21.4 ± 8.6	26.4 ± 8.8	17.9 ± 6.6	< 0.001
E wave velocity, cm/s	80 ± 16	84 ± 19	77 ± 13	0.027
A wave velocity, cm/s	64 ± 14	68 ± 16	61 ± 12	0.009
Deceleration time, ms	205 ± 36	208 ± 41	202 ± 32	0.384
Septal e’ velocity, cm/s	7.1 ± 1.7	6.4 ± 1.7	7.5 ± 1.6	< 0.001
Septal a’ velocity, cm/s	9.5 ± 2.1	9.3 ± 2.1	9.7 ± 2.0	< 0.001
Lateral e’ velocity, cm/s	10.0 ± 2.6	8.2 ± 2.0	11.1 ± 2.1	0.027
Lateral a’ velocity, cm/s	9.6 ± 2.2	10.6 ± 2.5	8.9 ± 1.8	0.009
E/e’ (septal)	12.2 ± 5.2	14.5 ± 6.4	10.7 ± 3.5	< 0.001

*BMI* body mass index, *LVESD* left ventricular end-systolic dimension, *LVEDD* left ventricular end-diastolic dimension, *LVEF* left ventricular ejection fraction, *LVSWT* left ventricular septal wall thickness, *LVPWT* left ventricular posterior wall thickness, *LA* left atrium.

^*^significance from the result of comparison between concordance and discordance groups.

### Comparisons between concordance and discordance groups

The results of comparative analyses between the concordance and discordance groups are also shown in Table 1. Subjects in the concordance group were older (59.6 ± 14.1 versus 53.5 ± 12.0 years, *p* = 0.012). Otherwise, there were no significance differences in terms of cardiovascular risk factors including sex, body mass index, hypertension, diabetes and dyslipidemia between the 2 groups (*p* > 0.05 for each). LV end-diastolic (LVEDD) and end-systolic dimensions (LVESD) were greater for the concordance group than those of discordance group (*p* < 0.05 for each). LAVI was significantly higher for the concordance group than that for the discordance group (26.4 ± 8.8 versus 17.9 ± 6.6 ml/m^2^, *p* < 0.001). Septal e’ wave velocity was significantly lower (6.4 ± 1.7 versus 7.5 ± 1.6 cm/s, *p* < 0.001) and E/e’ ratios were significantly higher (14.5 ± 6.4 versus 10.7 ± 3.5, *p* < 0.001) in the concordance groups than those in the discordance group.

### The value of lateral e’/a’ for discrimination of normal from grade II diastolic dysfunction

**Figure 3:**
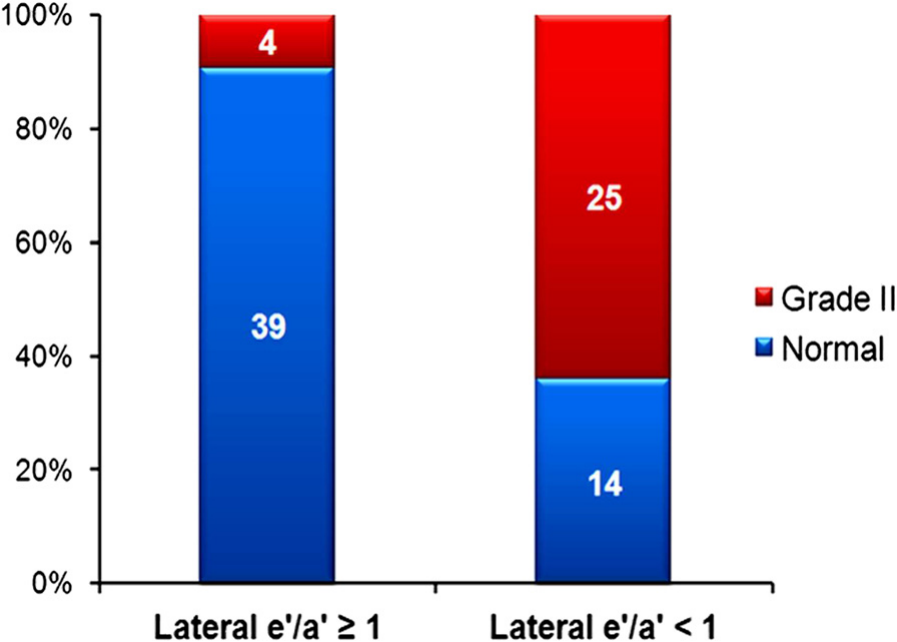
Distribution of normal and grade II diastolic dysfunction according to lateral tissue Doppler imaging patterns.

According to current guidelines [10], study subjects were grouped into normal and grade II diastolic dysfunction. If big discrepancies existed between parameters for diastolic dysfunction grading, this group of subjects was regarded as undetermined. The number of subjects in the normal, grade II and undetermined groups was 53 (43%), 29 (24%) and 40 (33%), respectively. Among subjects with lateral e’/a’ ≥ 1, only 4 subjects (9.3%) had grade II diastolic dysfunction, whereas among subjects with lateral e’/a’ < 1, the majority of subjects (n = 25, 64.1%) had grade II diastolic dysfunction (*p* < 0.001) (Figure 3).

### Independent association between LAVI and lateral e’/a’

**Figure 4:**
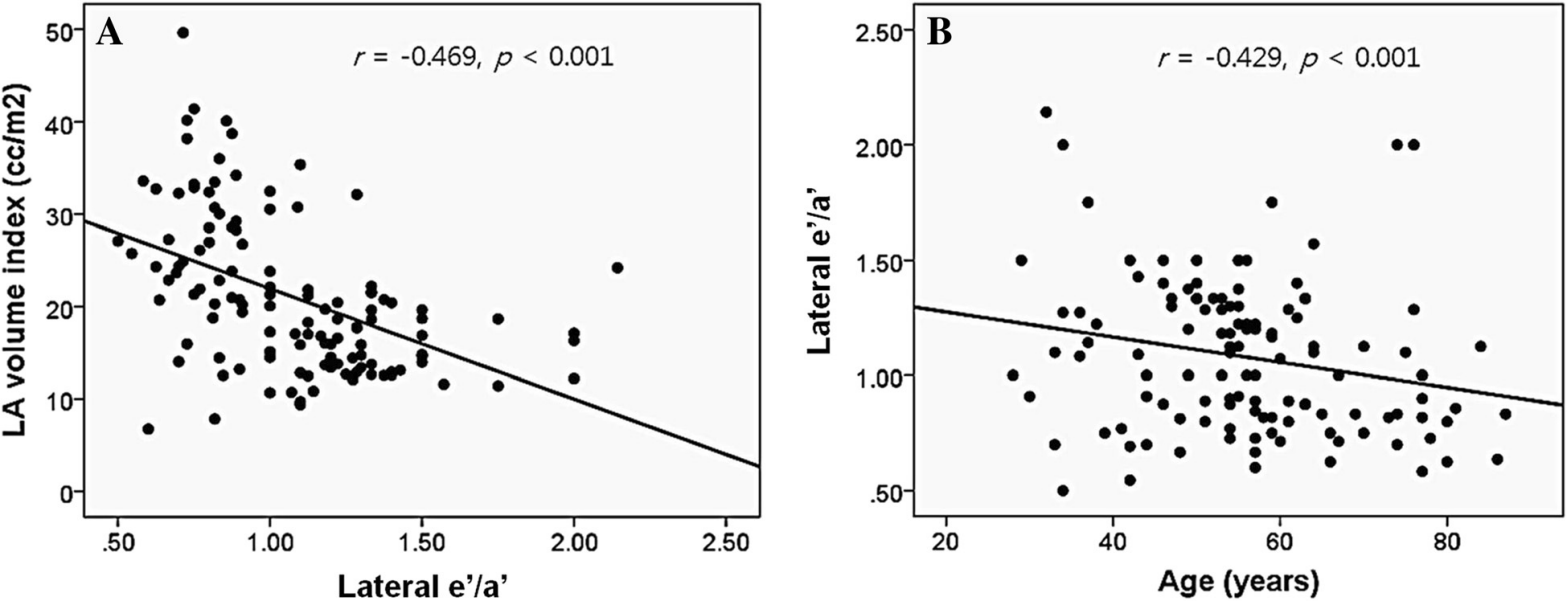
**Scatter plots showing the associations of lateral e’/a’ with left atrial volume index (A) and age (B).** LA, left atrium. *r*, correlation coefficient.

LAVI is a useful indicator of LV diastolic function [12]. We tested whether lateral e’/a’ had an association with LAVI. In a simple correlation analysis, lateral e’/a’ was significantly associated with LAVI: increases in lateral e’/a’ was associated with decreases in LAVI (*r* = −0.469, *p* < 0.001) (Figure 4A). It is well known that Doppler tissue e’ velocity decreases with age [13]. Therefore, age was a powerful confounder, and this was addressed in this study. As expected, lateral e’/a’ had a significant correlation with age, and decreased with advancing age (*r* = −0.429, *p* < 0.001) (Figure 4B).

**Figure 5:**
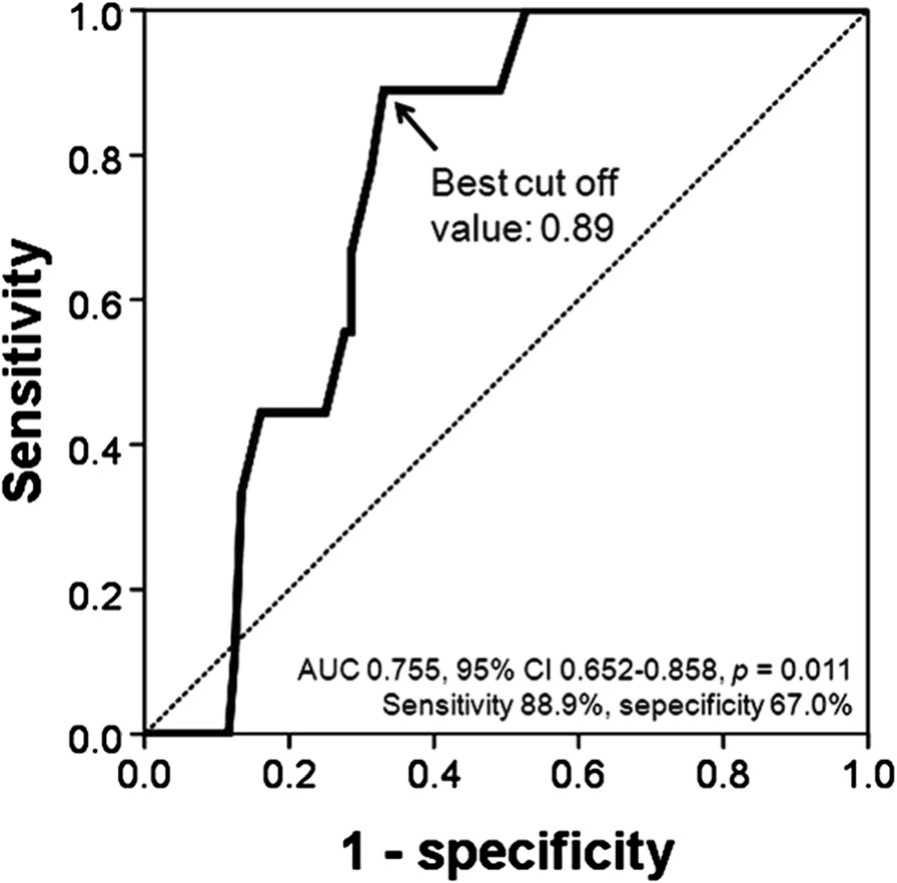
**Receiver operating characteristic curve analysis showing the value of lateral e’/a’ predicting left atrial volume index ≥ 34 ml/m^2^.** AUC, area under curve; CI, confidence interval.

Subsequently, potential confounders, including age, were addressed using a multivariable model. A multiple linear regression analysis showed that lateral e’/a’ was independently associated with LAVI (*β* = −0.484, *p* < 0.001), even after adjusting for potential confounders including age, sex, body mass index, hypertension and diabetes (Table 2). In the ROC curve analysis, the sensitivity and specificity for detection of LAVI ≥ 34 ml/m^2^ were 88.9% and 67.0%, respectively, with lateral e’/a’ of 0.89 as the best cut-off value (Figure 5).

**Table 2:** Independent associations between left atrial volume index and variables

**Variable**	***β***	**t**	***p***
Age	0.349	2.892	0.006
Female sex	0.021	0.189	0.850
Body mass index	0.248	2.086	0.042
Diabetes	0.136	1.245	0.219
Hypertension	0.013	0.115	0.909
Lateral e’/a’	−0.484	−4.677	< 0.001

Lateral e’ has been known to be a maker of diastolic dysfunction [10]. When the data was analyzed using lateral e’ velocity instead of lateral e’/a’, the lateral e’ velocity was significantly lower in subjects with grade II diastolic dysfunction than that in those with normal diastolic function (7.4 ± 1.9 versus 11.6 ± 2.0 cm/s, *p* < 0.001). Lateral e’ velocity was also negatively associated with LAVI even after adjusting for potential confounders (*β* = −0.360, *p* = 0.011).

The data was analyzed for any correlations between lateral e’/a’ and LAVI in subjects with normal diastolic function (n = 263) and grade I diastolic dysfunction (relaxation abnormality, n = 276). In the univariate analysis, there was a significant correlation between lateral e’/a’ and LAVI in subjects with normal diastolic function (*r* = −0.348, *p* < 0.001), but this was not an independent association as it was not significant in the subsequent multivariable analysis (*β* = −0.175, *p* = 0.086). There were no significant correlations between lateral e’/a’ and LAVI in subjects with grad I diastolic dysfunction in both univariate and multivariable analyses.

## Discussion

The present study identified 2 distinct groups of lateral TDI patterns among relatively healthy subjects without structural problems of the heart but with pseudonormal pattern of mitral inflow. Subjects with a concordance pattern between septal and lateral TDI were older, and had more unfavorable echocardiographic parameters including high LAVI and E/e’, and demonstrated more advanced LV diastolic dysfunction. On the other hand, subjects with a discordance pattern showed nearly normal or mild diastolic dysfunction. Lateral e’/a’ had an independent correlation with LAVI even after controlling for potential confounders such as age, sex, body mass index, hypertension and diabetes. This suggests that lateral e’/a’ would be a valuable parameter for assessing diastolic function in subjects with a pseudonormal pattern of mitral inflow.

To date, subjects with pseudonormal pattern of mitral inflow has not been further stratified. These subjects are often considered to have advanced diastolic dysfunction, but the present study have identified that more than half of these subjects (72/122 = 59%) have nearly normal diastolic function with small LAVI and low E/e’. We believe that this group of subjects may represent those with “early diastolic dysfunction”.

Many different echocardiographic parameters exist in the assessment of diastolic function, and they can help differentiate between normal and pseudonormal patterns of diastolic function [9]. However, each test has limitations such as dependency on loading conditions and image quality [9]. In addition, obtaining appropriate hemodynamic maneuver to differentiate normal and pseudonormal diastolic dysfunctions may be difficult to achieve in all subjects. Moreover, application of several tests is not an easy task in busy clinical settings. In this regard, checking lateral TDI patterns seems to be a simple and useful tool for the diagnosis of LV diastolic function. Measurement of TDI of mitral annulus has been known to be relatively load independent [5] and can be performed in nearly all subjects (95%), regardless of image quality [9]. In addition, it does not require training of hemodynamic maneuvers to patients or sonographers. From the study results, we can suggest a possible diagnostic pathway for the assessment of LV diastolic function. If a healthy subject without significant cardiac structural abnormality has a pseudonormal pattern of mitral inflow, lateral TDI evaluation can be helpful. With lateral e’/a’ ≥ 1, a subject can be considered roughly to have no significant diastolic dysfunction but an early diastolic dysfunction.

Our results imply that septal e’/a’ is more sensitive marker of diastolic dysfunction compared to lateral e’/a’. Septal e’/a’ reversed in the early stage of diastolic dysfunction, however lateral e’/a’ dose not reverse until a more advanced stage of diastolic dysfunction develops. These findings are in line with those of a previous report [8]. Consequently, the severity of diastolic dysfunction is often overestimated especially in subjects with E/A ≥ 1 and septal e’/a’ < 1 unless further tests are performed [14]. Therefore, combination of septal and lateral e’/a’ is useful for the discrimination of early stage of diastolic dysfunction from more advanced stages of diastolic dysfunction among patients with mitral inflow patterns of E/A ≥ 1.

In order to assess the role of lateral e’/a’ as a discriminator of diastolic dysfunction, LAVI was used as a reference standard of LV diastolic dysfunction. As commonly observed with TDI, there is an inverse correlation between age and e’ velocity and e’/a’ [5,8,13,15], and this correlation was also observed in our study (Figure 3). However, we noted that an independent association between lateral e’/a’ and LAVI persisted even when adjusting for confounders including age and other risk factors in a multivariable model.

Because there are various sites for obtaining TDI, a variety of cut-off values for the diagnosis of diastolic dysfunction and/or high LV filling pressure have been suggested. Sohn et al. reported that septal e’ < 8.5 cm/s and e’/a’ < 1 discriminated a pseudonormal pattern from a normal pattern with a sensitivity of 88% and a specificity of 67% [5]. A study by Nagueh et al. demonstrated that lateral E/e’ > 10 correlated well with elevated LV filling pressure [16]. Ommen and colleagues reported that E/e’ < 8 predicted normal LV end-diastolic pressure, and E/e’ > 15 identified increased LV end-diastolic pressure [7]. Several studies have performed compared the diagnostic utility of TDI at different examination locations. Srivastava et al. compared septal versus lateral TDI and found that septal e’ provides better diagnostic performance [17]. In a study of healthy subjects with normal LV systolic function, Firstenberg et al. reported a good correlation between septal e’ velocities and LV filling pressures as estimated by invasive hemodynamic study, whereas lateral e’ did not show the same correlation [18]. Park and colleagues showed that septal TDI tends to overestimate the severity of LV diastolic dysfunction when compared with lateral TDI, and suggested that lateral TDI measurements more accurately reflects LV diastolic dysfunction than septal TDI measurement do [14]. Other studies have also reported that lateral TDI correlates best with LV filling pressure and with indices of LV stiffness in subjects with normal ejection fraction or coronary artery disease [19-21]. Current guidelines recommend the use of average e’ obtained from septal and lateral sides of the mitral annulus for prediction of LV filling pressure [10]. Taken together, to date, there has been no consensus on appropriate selection of annulus for TDI assessment of diastolic function and filling pressure.

### Study limitations

The study sample size is small. Enrolled subjects were healthy without cardiac structural abnormalities, and therefore, the results of the study cannot be generalized to other populaitons. The most widely used reference parameter for diastolic function is LV relaxation time; however, LV catheterization was not performed in this study. Other parameters which can be measured during echocardiography, such as mitral inflow pattern with the Valsalva maneuver, pulmonary venous flow pattern and color M-mode propagation velocity, were not evaluated in this study.

## Conclusions

For subjects with E/A ≥ 1 and septal e’/a’ < 1, the severity of diastolic dysfunction is often overestimated. In this case, lateral TDI evaluation is helpful for assessment of diastolic dysfunction. Lateral e’/a’ ≥ 1 is a valuable indicator of early diastolic dysfunction but not for advanced diastolic dysfunction in this group of people.

## Abbreviations

DT: Deceleration time; HFpEF: Heart failure with preserved ejection fraction; LA: Left atrium; LAVI: Left atrial volume index; LV: Left ventricle; LVEDD: Left ventricular end-diastolic dimension; LVESD: Left ventricular end-systolic dimension; SD: Standard deviation; ROC: Receiver operating characteristics; TDI: Tissue Doppler imaging; TTE: Transthoracic echocardiography.

## Competing interests

The authors declare that they have no competing interests.

## Authors’ contributions

The work presented here was carried out in collaboration between all authors. H-LK analyzed data and wrote the paper; J-HZ designed the study, interpreted the results, and had all responsibilities of this work; J-BS, Y-JK, W-YC, S-HK, M-AK and D-WS were involved in study design and interpretation of data. All authors read and approved the final manuscript.
